# Drill-guided femoral nail extraction: a novel technique

**DOI:** 10.1093/jscr/rjab523

**Published:** 2021-11-29

**Authors:** Alexandros I Misailidis, Ioannis D Aifantis, Dimitrios Pallis, Pantelis K Mitsikostas, Georgios Gourtzelidis, Konstantinos Tsivelekas, Stamatios A Papadakis

**Affiliations:** B' Department of Orthopaedics, KAT General Hospital of Attica, Kifisia, Greece; B' Department of Orthopaedics, KAT General Hospital of Attica, Kifisia, Greece; B' Department of Orthopaedics, KAT General Hospital of Attica, Kifisia, Greece; B' Department of Orthopaedics, KAT General Hospital of Attica, Kifisia, Greece; B' Department of Orthopaedics, KAT General Hospital of Attica, Kifisia, Greece; B' Department of Orthopaedics, KAT General Hospital of Attica, Kifisia, Greece; B' Department of Orthopaedics, KAT General Hospital of Attica, Kifisia, Greece

## Abstract

Femoral nail extraction, although it is considered a challenging procedure for orthopedic surgeons, can be simplified. We present a new technique to aid the removal of a proximally (antegrade) inserted femoral nail by applying drilling consecutively in order to identify the margins and depth of the nail into the intramedyllary canal of the femur. The damage to the bone is minimal as we use k-wires or drilling and in our practice was uneventful. This technique is the first to be reported in literature. Most authors suggest techniques that enable radiolucent table and fluoroscopy techniques using C-arm. With this technique, traction table and fluoroscopy techniques seem to be less essential to accomplish the removal of a proximally (antegrade) inserted femoral nail.

## INTRODUCTION

Femoral nail extraction is a challenging procedure for orthopedic surgeons. We present a novel technique, which can be used mainly in cases of antegrade femoral intramedullary nail subsidence, and in the presence of heterotopic bone formation. Techniques that are presented in literature involve fluoroscopy and percutaneous identification of the intramedullary canal. Apart from the insertion point, another challenge is the incision area. It may be different when the nail is placed in a supine position and when extracted with the patient in lateral decubitus position. All these techniques try to minimize the soft tissue and bone damage by being more precise as far as the skin incision and the tip of the nail is concerned.

## CASE PRESENTATION

Four patients suffering of femoral shaft fractures were treated with antegrade intramedullary femoral nail. There were three males and one female with a mean age of 37.2 years old (range 22–47). Three patients were treated with Smith and Nephew Triger TAN FAN femoral nail and one with a Stryker T2 antegrade nail. All these patients underwent a femoral nail removal with a novel technique, between 20 and 26 months post injury. Post-operative course of the patients was uneventful. The study was approved by the Scientific Committee of our institution.

### Surgical technique

**Figure 1:**
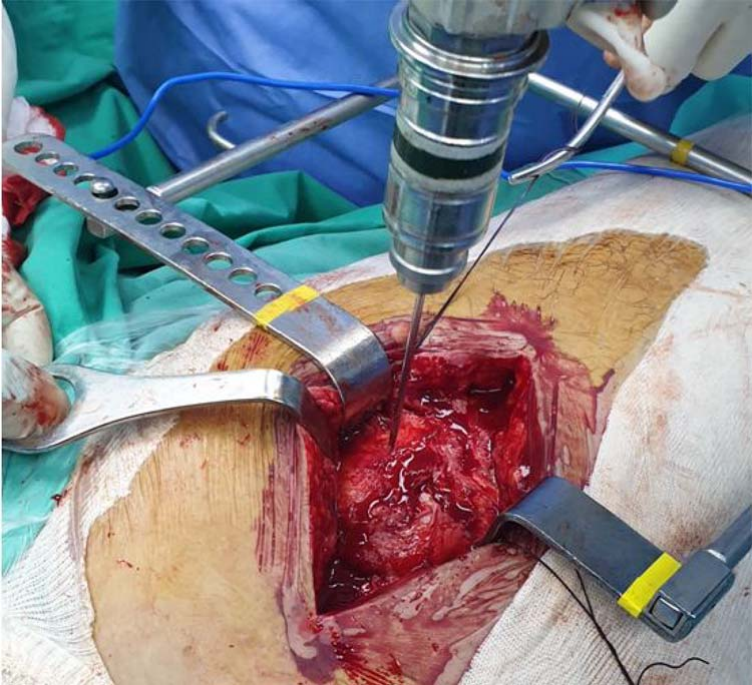
Intraoperative image showing the position of a k-wire in order to indentify the anterior margin of the intramedullary nail.

**Figure 2:**
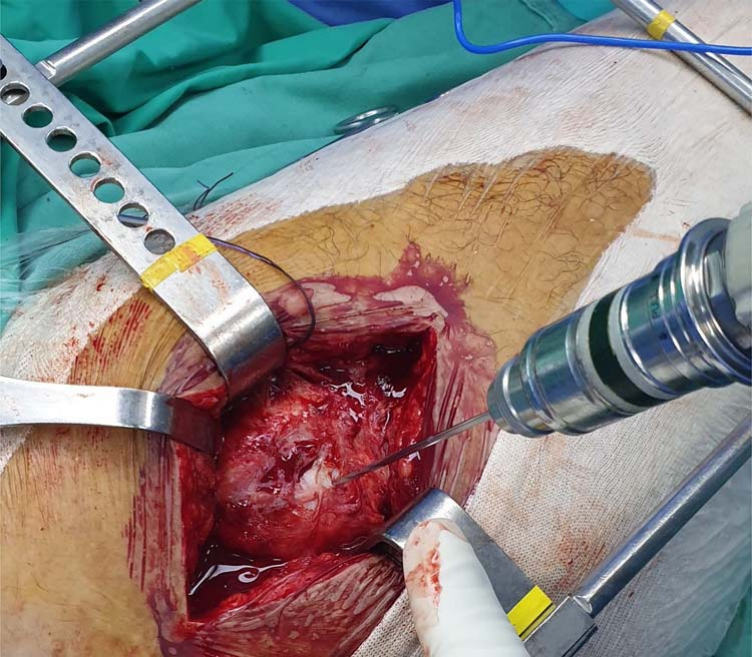
Intraoperative image showing the position of a k-wire in order to indentify the posterior margin of the intramedullary nail.

**Figure 3:**
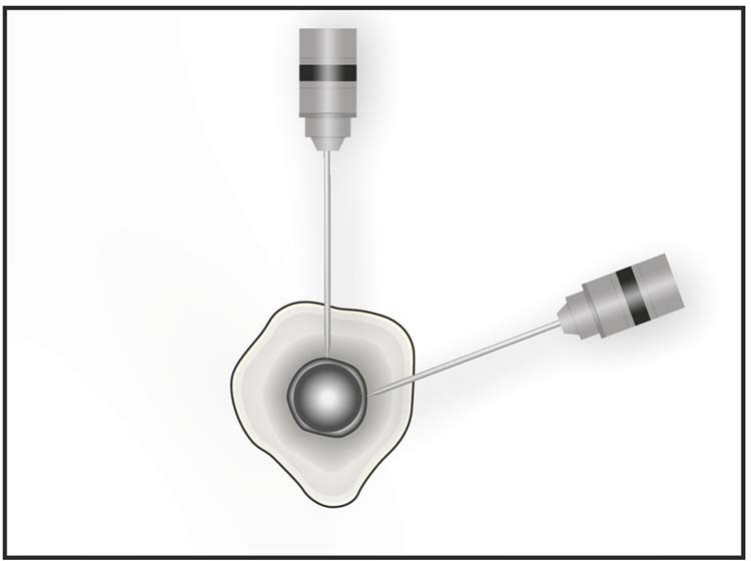
Image showing the metal on metal effect that is produced when the k-wire has reached the outer surface of the nail in different planes.

**Figure 4:**
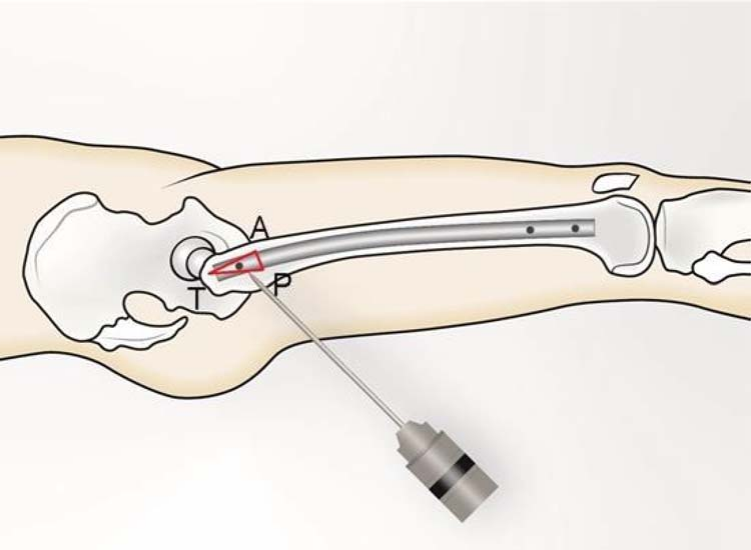
(TAP), is the triangle formed (in red). (T) represents the proximal tip of the intramedullary nail; (A) the anterior margin of the intramedullary nail and (P) the posterior margin of the intramedullary nail.

The patient is placed in lateral decubitus position. There is no need of a traction table and fluoroscopy in order to identify the intramedullary canal and the nail inserted. Palpating the prominence of the greater trochanter on the lateral side, an incision is made over it and can be extended distally and proximally. The fasciae latae is carefully incised. The femoral diaphysis and the origin of vastus lateralis are identified. Above the origin of vastus lateralis, k-wires are drilled in order to identify, the anterior and posterior margins of the intramedullary nail in the sagittal plane and mark them and can be left in place (Figs 1 and 2). This is easily performed due to the metal-on-metal effect that is produced during drilling (Fig. 3). Knowing the anterior (point A) and posterior (point P) margins of the intramedullary nail, multiple drillings are performed proximally and perpendicular to the median of the (AP) side of the formed triangle (TAP). The drilling point where no metal-on-metal effect is produced corresponds to the proximal endpoint of the intramedullary nail (point T). Those points form a triangle (TAP) on the lateral side of the femur with the top (T) highlighting the endpoint of the intramedullary nail, and the sides the anterior (A) and posterior (P) margins (Fig. 4). Drilling with k-wires also helps us determine the depth of the nail.

In an open procedure, this axis is a good landmark for entry point identification. With this method, it is possible to measure the depth of the nail and calculate the bone and the ossified tissue that needs to be removed. A reamer is used to create a round hole above the area of the tip of the greater trochanter until the end cup arises [1]. The end cup is removed and the extractor is attached. Percutaneous removal of embedded distal locking screws can be also challenging and may require a bigger incision. After removing the distal screw, the nail can finally be extracted.

## DISCUSSION

Minimally invasive techniques for intramedullary nail removal are not a new practice. Most authors suggest techniques that enable radiolucent table and fluoroscopy techniques using C-arm. As a common practice, the patient is placed in a supine position with the aid of traction on a fracture table. Fluoroscopy is an indispensable part of this process, in order to identify the exact position and the depth of the nail. Guided drilling with a cannulated drill secures minimal soft tissue and bone damage at the area of the greater trochanter [2–4]. Despite all of that, heterotopic ossification may elongate significantly the operation time while impeding adequate exposure of the nail. Specific instrumentation involving cannulated soft tissue protectors and curettes seem to be helpful for the skin protection and bone and soft tissue debridement [2].

Positioning of the patient when intramedullary nail extraction is performed may be different from the one when the nail is placed. Fluoroscopy technique routinely is used with the patient in supine position, while its use is restricted with the patient in lateral decubitus position. Femoral nailing can be carried out with the patient either supine or in lateral decubitus position. For all these reasons, the primary skin incision may vary from the incision needed for the nail extraction. To overcome this, Wood suggests frontal and lateral fluoroscopic images to be taken with use of a guidewire placed over the skin in an attempt to identify the femoral nail. The combinations of these two lines intersect at the entrance point of the extractor [5].

**Figure 5:**
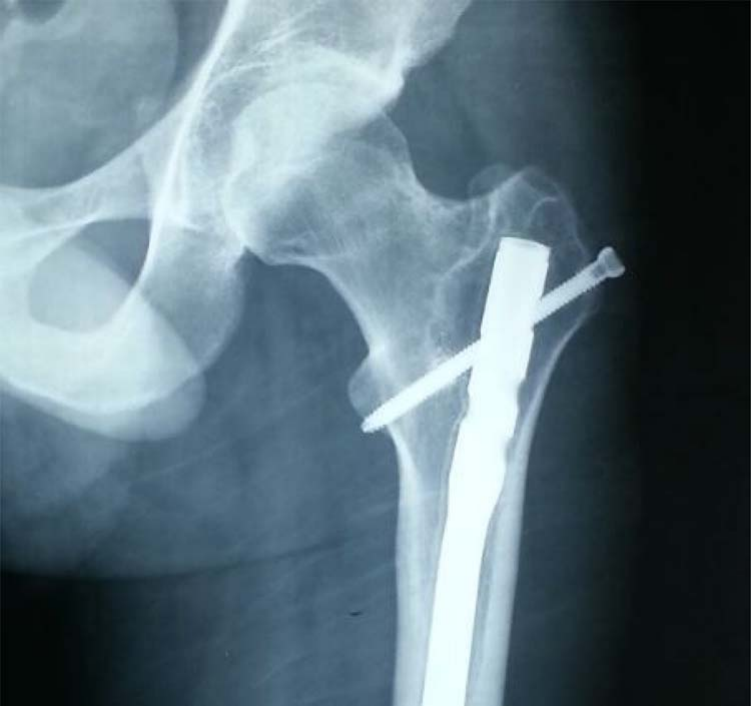
Anteroposterior radiograph showing a buried intramedullary nail.

**Figure 6:**
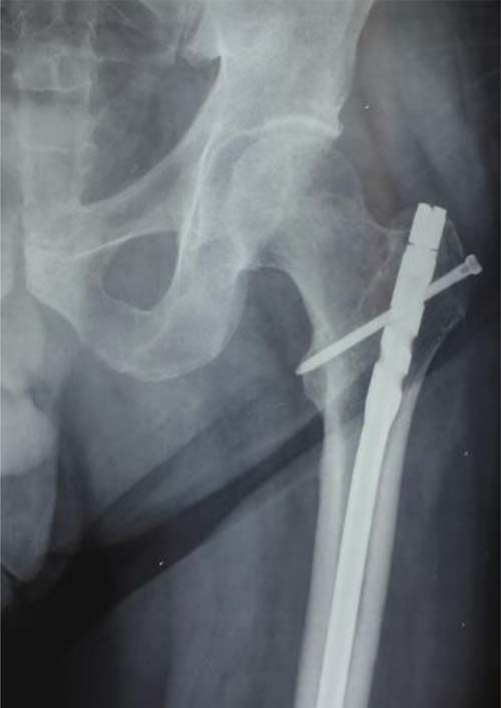
Anteroposterior radiograph showing the tip of the intramedullary nail right at the level of the greater trochanter.

According to our practice, a procedure as demanding as intramedullary nail extraction can be simplified. The instrumentation required is limited by the lower level or no usage of fluoroscopy. Radiation exposure time for the patient and the surgeon is decreased. There is no necessity for radiolucent table and traction, even though they may be helpful. Moreover, fewer restrictions arise for the patient position during the procedure. Despite the fact that lateral decubitus position is mostly preferred, both lateral decubitus and supine positions are acceptable for this technique. In our opinion, this technique is useful mainly in cases of buried or subsiding nails (Fig. 5). It can be also useful in the presence of heterotopic bone formation. In cases where the nail is at the level of the greater trochanter (Fig. 6), the technique can be also helpful as it is difficult to find the tip of the nail because of the soft tissue envelope and the associated scar formation.

Lovell *et al.* published a technique for the extraction of a broken femoral nail, and they also made a review of the relative strengths and weaknesses of current published techniques [6]. Based on their findings and their review report, this technique can be differentiated from other methods as follows: it is of low cost and does not require fluoroscopy. It is simple to perform and does not require special equipment. Furthermore, it does not require special skills and can also be used to extract femoral and tibial nails either narrow or solid. It is also useful in cases with buried nails or in the presence of heterotopic formation and uses original wounds without compromising the soft tissue envelope.

One of our concerns about this method is the potential bone weakening. In our opinion, cortical bone weakening due to multiple drilling is minimal and should not be taken into consideration. In the hands of a trained practitioner, this constraint can undoubtedly be overcome. Post-operative course in our patients was uneventful. Intraoperative complication rates concerning soft tissue damage, bone exposure, greater trochanter post-operative durability, abductor muscles functionality and iatrogenic neurovascular injuries have been described in current literature [7–10]. None of these complications were seen in our patients. However, the number of our cases that this novel technique was applied to was small.
